# Comparison of Home-Based vs Center-Based Cardiac Rehabilitation in Hospitalization, Medication Adherence, and Risk Factor Control Among Patients With Cardiovascular Disease

**DOI:** 10.1001/jamanetworkopen.2022.28720

**Published:** 2022-08-25

**Authors:** Chileshe Nkonde-Price, Kristi Reynolds, Michael Najem, Su-Jau Yang, Columbus Batiste, Timothy Cotter, Debora Lahti, Nancy Gin, Tadashi Funahashi

**Affiliations:** 1 Department of Cardiology, Kaiser Permanente West Los Angeles Medical Center, Los Angeles, California; 2 Department of Clinical Science, Kaiser Permanente Bernard J. Tyson School of Medicine, Pasadena, California; 3 Department of Research & Evaluation, Kaiser Permanente Southern California, Pasadena; 4 Southern California Permanente Medical Group, Pasadena; 5 Department of Health Systems Science, Kaiser Permanente Bernard J. Tyson School of Medicine, Pasadena, California; 6 Department of Cardiology, Kaiser Permanente Riverside Medical Center, Los Angeles, California; 7 Kaiser Permanente Center for Health Innovation, Tustin, California; 8 Department of Cardiology, Kaiser Permanente Baldwin Park Medical Center, Los Angeles, California; 9 Department of Orthopedic Surgery, Kaiser Permanente Orange County Medical Center, Anaheim, California

## Abstract

**Importance:**

Prior studies have suggested that participation in home-based cardiac rehabilitation (HBCR) vs center-based cardiac rehabilitation (CBCR) results in similar clinical outcomes in patients with low to moderate risk; however, outcome data from demographically diverse populations and patients who are medically complex are lacking.

**Objective:**

To compare hospitalizations, medication adherence, and cardiovascular risk factor control between participants in HBCR vs CBCR.

**Design, Setting, and Participants:**

This retrospective cohort study was conducted among patients in Kaiser Permanente Southern California (KPSC), an integrated health care system serving approximately 4.7 million patients, who participated in CR between April 1, 2018, and April 30, 2019, and with follow-up through April 30, 2020. Data were analyzed from January 2021 to January 2022.

**Exposures:**

Participation in 1 or more HBCR or CBCR sessions.

**Main Outcomes and Measures:**

The primary outcome was 12-month all-cause hospitalization. Secondary outcomes included all-cause hospitalizations at 30 and 90 days; 30-day, 90-day, and 12-month cardiovascular hospitalizations; and medication adherence and cardiovascular risk factor control at 12 months. Logistic regression was used to compare hospitalization, medication adherence, and cardiovascular risk factor control, with inverse probability treatment weighting (IPTW) to adjust for demographic and clinical characteristics.

**Results:**

Of 2556 patients who participated in CR (mean [SD] age, 66.7 [11.2] years; 754 [29.5%] women; 1196 participants [46.8%] with Charlson Comorbidity Index ≥4), there were 289 Asian or Pacific Islander patients (11.3%), 193 Black patients (7.6%), 611 Hispanic patients (23.9%), and 1419 White patients (55.5%). A total of 1241 participants (48.5%) received HBCR and 1315 participants (51.5%) received CBCR. After IPTW, patients who received HBCR had lower odds of hospitalization at 12 months (odds ratio [OR], 0.79; 95% CI, 0.64-0.97) but similar odds of adherence to β-blockers (OR, 1.18; 95% CI, 0.98-1.42) and statins (OR, 1.02; 95% CI, 0.84-1.25) and of control of blood pressure (OR, 0.98; 95% CI, 0.81-1.17), low-density lipoprotein cholesterol (OR, 0.98; 95% CI, 0.81-1.20), and hemoglobin A_1c_ (OR, 0.98; 95% CI, 0.82-1.18) at 12 months compared with patients who received CBCR.

**Conclusions and Relevance:**

These findings suggest that HBCR in a demographically diverse population, including patients with high risk who are medically complex, was associated with fewer hospitalizations at 12 months compared with patients who participated in CBCR. This study strengthens the evidence supporting HBCR in previously understudied patient populations.

## Key Points

**Question:**

What are the associations of home-based vs center-based cardiac rehabilitation (CR) with hospitalizations, medication adherence, and cardiovascular risk factor control among diverse patients with cardiovascular disease and high risk?

**Findings:**

In this cohort study of 2556 demographically diverse patients with high risk in a large integrated health system, there were fewer hospitalizations at 12 months among participants in home-based CR compared with participants in center-based CR programs.

**Meaning:**

This cohort study found that participation in home-based CR was associated with fewer hospitalizations at 12 months compared with participation in center-based CR.

## Teaser

This cohort study compares hospitalizations, medication adherence, and cardiovascular risk factor control between patients participating in home-based vs center-based cardiac rehabilitation.

## Introduction

Cardiac rehabilitation (CR) performed in the hospital-based setting is a well-studied, effective intervention known to improve health outcomes in patients with cardiovascular disease (CVD).^1,2,3,4,5^ Guidelines recommend CR in patients after myocardial infarction (MI),^6,7^ cardiac surgery,^8,9^ and elective percutaneous coronary intervention (PCI) and in patients with stable angina or stable heart failure.^10,11^ Despite this recommendation, studies have shown that more than 80% of eligible patients in the US do not participate in CR,^12^ with the lowest participation rates in women, members of racial and ethnic minority groups (eg, Black or African American, Hispanic or Latino, Asian, Native American or Alaska Native, and Native Hawaiian or Pacific Islander), and individuals with multiple comorbidities.^12,13,14,15,16^ CR performed in the nonhospital setting, such as home-based CR (HBCR) is an alternative strategy that has been developed to increase participation in all eligible patient populations.

Prior randomized clinical trials (RCTs) have compared outcomes among selected patients with low to moderate risk who are clinically stable and have consistently found similar clinical outcomes among patients who participate in HBCR vs center-based CR (CBCR).^17,18,19^ Additionally, the Cochrane collaborative has conducted 3 meta-analyses^1,20,21^ that have each combined RCTs of HBCR vs CBCR and consistently found that there is there low- to moderate-strength evidence that HBCR and CBCR are associated with similar outcomes in the selected patients with low to moderate risk who are clinically stable enrolled in these trials. Based on this evidence, the American Heart Association has endorsed HBCR as a reasonable option for these patients but has highlighted that data are lacking in demographically diverse populations (ie, women and racial and ethnic minorities) and patients with higher risk (ie, patients with multiple comorbidities).^22^

To address these knowledge gaps, we aimed to compare 12-month hospitalizations, medication adherence, and CVD risk factor control in a large demographically diverse population that included patients with higher risk and who were medically complex and who participated in HBCR or CBCR programs in a large, integrated health care system in Southern California.

## Methods

This cohort study was approved by the institutional review board at Kaiser Permanente Southern California (KPSC). A waiver of informed consent was obtained because of the nature of this retrospective, data-only study. This study followed the Strengthening the Reporting of Observational Studies in Epidemiology (STROBE) reporting guideline.

### Study Design and Data Source

We conducted a retrospective observational cohort study in KPSC, an integrated health care system serving approximately 4.7 million patients. KPSC provides care to a racially, ethnically, and socioeconomically diverse population broadly representative of the racial and ethnic groups in Southern California.^23^ Comprehensive information on the medical care KPSC patients receive is prospectively captured through an electronic medical record (EMR). The Kaiser Permanente Virtual Data Warehouse and EMR were the primary data sources for patient identification and characterization. The Virtual Data Warehouse includes EMR-based data sets with linked sociodemographic, administrative, pharmacy, laboratory results, and health care use data.^24,25^

### Setting and Participants

For this study, we identified patients who participated in CR between April 1, 2018, and April 30, 2019. Consistent with previous observational studies, patients were classified as having received CR if they had completed at least 1 CR session.^15,26,27^ To be included in the study, patients must have experienced more than 1 of the following Center for Medicaid & Medicare Services CR-eligible cardiovascular events^28,29^: acute MI, stable angina, elective PCI, chronic heart failure (CHF) or cardiothoracic surgical treatment (heart or heart-lung transplantation, coronary artery bypass graft [CABG], valve repair or replacement) within 30 days before their first CR session. Events were identified based on *International Statistical Classification of Diseases, Tenth Revision, Clinical Modification (ICD-10-CM)* codes. All events were verified by EMR review performed by 2 of us (C.N.-P. and M.N.). Patients with multiple CR-eligible events within 30 days before CR participation were reclassified according to the most severe diagnosis using the following established rankings for CVD diagnosis and cardiothoracic surgical treatment.^26^ For CVD diagnosis, the ranking was (1) ST-segment elevation MI, (2) non–ST-segment elevation MI, (3) elective PCI, (4) stable angina, and (5) CHF. For cardiothoracic surgical treatment, the ranking was (1) heart or heart-lung transplantation, (2) CABG, and (3) valve repair or replacement. We excluded 145 patients without a CR-eligible diagnosis, 2 patients younger than 18 years, 426 patients with less than 12 months of continuous KPSC membership before and after CR participation, and 9 patients who moved outside of California in the follow-up period. The derivation of the study cohort is presented in the eFigure in the Supplement.

### Exposure

Participation in 1 or more HBCR or CBCR sessions was the primary exposure. Details of the KPSC HBCR Program have been previously described.^30^ Briefly, the program is a technology-enabled (mobile phone application linked to a wearable smartwatch, which was standardized for all participants) 8-week comprehensive CR program that consists of (1) unsupervised exercise sessions, (2) weekly CR nurse phone calls, and (3) health education (eTable 1 in the Supplement). The comprehensive structure (exercise training, telephone support, and health education) is similar to the structure used in most of the RCTs (14 of 23 studies) included in the Cochrane collaborative HBCR vs CBCR meta-analysis.^1^

CBCR was performed in multiple centers across the KPSC catchment area. All CBCR centers were accredited by the American Association of Cardiovascular and Pulmonary Rehabilitation. The decision to refer a patient to HBCR vs CBCR was at the discretion of the treating cardiologist.

An HBCR session was defined as completion of at least 1 exercise session, recorded by the smartwatch. A CBCR session was defined using outpatient claims data. Specifically, the presence of at least 1 Healthcare Common Procedure Coding System code for physician services for outpatient CR without (code 93797) or with (code 93798) continuous electrocardiographic monitoring or intensive CR with or without continuous electrocardiographic monitoring and with (code G0422) or without (code G0423) exercise in combination with a place of service code of 11 (office), 19 (off-campus outpatient hospital), or 22 (on-campus outpatient hospital).^15^

### Covariates

Demographics (age, sex, race and ethnicity, need for interpreter services, geocoded median household income, and marital status) were collected from the EMR. Self-reported race and ethnicity were categorized as Asian or Pacific Islander, Black, Hispanic, White, and other, including individuals listed as American Indian or Alaska Native, more than 1 race, other race, or unknown race. Given that race and ethnicity are social constructs without scientific or biological meaning, we report race and ethnicity alongside other sociodemographic variables in order to evaluate racism, the realities of social stratification, and injustices and inequities in health care.^31,32,33,34^ Comorbidities (hypertension, hyperlipidemia, diabetes, prior MI, CHF, chronic kidney disease [CKD], depression, and smoking status), medical complexity (evaluated using the Charlson Comorbidity Index^35^), reason for referral to CR, prior health care utilization (hospitalizations and emergency department and urgent care visits) and baseline cardiovascular risk factors (blood pressure, low-density lipoprotein [LDL]–cholesterol, hemoglobin A_1c_ [HbA_1c_], and body mass index [BMI; calculated as weight in kilograms divided by height in meters squared]) were identified from the EMR and billing claims for outside services in the 12 months prior to the index date. The index date was defined as date of enrollment in CR. For patients who had multiple measures over the 12-month period, we used the last measure, closest to the index date. Adherence, defined as total exercise sessions during CR, was recorded by the smartwatch and transmitted to the EMR for participants in HBCR and determined by billing claims for participants in CBCR.

### Outcome Measures

The primary outcome was 12-month all-cause hospitalization. Hospitalizations that occurred within the 12-month period after the index date were extracted from the EMR and billing claims from outside services. The principal diagnosis for each hospitalization was assessed using the primary *ICD-10-CM* code, which reflects the main reason for admission. The primary reason for hospitalization was divided into all-cause (all *ICD-10-CM* codes) or cardiovascular (*ICD-10-CM* codes I00-I99). Secondary outcomes included hospitalizations at 30 and 90 days, as well as medication adherence and cardiovascular risk factor control at 12 months.

Medication information within the 12-month period after the index date was extracted from the outpatient pharmacy dispensing record. We calculated the proportion of days covered (PDC) over the entire 12-month period using dates and days of supply of the prescription filled. Adherence was classified as high (PDC ≥80%), partial (PDC 40% to <80%), and low (PDC <40%).^36^

Cardiovascular risk factor control was examined in the 12 months after the index date using data extracted from the EMR. Risk factor control was defined as systolic blood pressure less than 140 mm Hg, diastolic blood pressure less than 90 mm Hg, LDL-cholesterol less than 100 mg/dL (to convert to millimoles per liter, multiply by 0.0259), HbA_1c_ less than 7% (to convert to proportion of total hemoglobin, multiply by 0.01), and BMI less than 25. For patients who had multiple measures over the 12-month follow up period, we used the last measure. Blood pressure values were available for all patients. Missing values for LDL and HbA_1c_ were imputed using hot deck imputation.

### Statistical Analysis

Baseline characteristics between CR groups were compared using χ^2^ test for categorical variables and *t* tests for continuous variables. Differences in characteristics between groups were balanced by performing propensity score analysis using the inverse probability treatment weighted (IPTW) approach.^37^ The probability of participating in the HBCR program for each patient was estimated by logistic regression model including the following baseline covariates: demographics (age, sex, race and ethnicity, marital status, geocoded median household income, interpreter service, ever smoker, hospitalization, and referral to CR program owing to cardiothoracic surgical treatment), comorbidities (Charlson Comorbidity Index, CKD, diabetes, hyperlipidemia, hypertension, CHF, MI, stroke, and depression), vital signs (BMI, systolic blood pressure, and diastolic blood pressure), and laboratory results (LDL cholesterol and HbA_1c_). The propensity score was calculated from the regression model for each patient. The weight was then calculated as the inverse of the propensity score for patients in the HBCR program and was the inverse of 1 minus the propensity score for patients in the CBCR program. The obtained weights were applied in statistical models to estimate the program associations with the outcomes.

Standardized differences were calculated to compare the participant baseline characteristics before and after weighting and to assess whether the IPTW achieved balance in the distribution of characteristics between the CR programs, with a value less than 0.1 indicating good balance or a negligible difference. Logistic regression models were used to estimate odds ratios (ORs) and 95% CIs of the outcomes. Tests were 2-tailed, with significance set at *P* < .05. To explore potential differences in outcomes among sex, racial and ethnic, and clinical risk subgroups, we tested for interactions by sex, race and ethnicity, and Charlson Comorbidity Index. All statistical analyses were performed in SAS, version 7.1 (SAS Institute). Data were analyzed from January 2021 to January 2022.

## Results

### Patient Characteristics

In this cohort of 2556 patients who participated in CR (mean [SD] age, 66.7 [11.2] years, 754 [29.5%] women; 1196 participants [46.8%] with Charlson Comorbidity Index ≥4), there were 289 Asian or Pacific Islander patients (11.3%), 193 Black patients (7.6%), 611 Hispanic patients (23.9%), and 1419 White patients (55.5%). A total of 1241 participants (48.5%) received HBCR and 1315 participants (51.5%) received CBCR (Table 1).

**Table 1.** Demographic and Clinical Characteristics of Patients Participating in CBCR and HBCR

Characteristic	Patients, No. (%)			*P* value
	Total (N = 2556)	CBCR (n = 1315)	HBCR (n = 1241)	
Age, y				
Mean (SD)	66.7 (11.2)	68.3 (10.8)	65.1 (11.3)	<.001
<45	99 (3.9)	33 (2.5)	66 (5.3)	<.001
45-65	918 (35.9)	423 (32.2)	495 (39.9)	
>65	1539 (60.2)	859 (65.3)	680 (54.8)	
Sex				
Women	754 (29.5)	372 (28.3)	382 (30.8)	.17
Men	1802 (70.5)	943 (71.7)	859 (69.2)	
Race and ethnicity				
Asian or Pacific Islander	289 (11.3)	158 (12.0)	131 (10.6)	.02
Black	193 (7.6)	90 (6.8)	103 (8.3)	
Hispanic	611 (23.9)	288 (11.3)	323 (26.0)	
White	1419 (55.5)	760 (57.8)	659 (53.1)	
Other^a^	44 (1.7)	19 (1.4)	25 (2.0)	
Language interpreter needed	142 (5.6)	82 (6.2)	60 (4.8)	.12
Neighborhood median household income, mean (SD), $	82 466 (31 885)	84902 (34 129)	79885 (29 113)	.002
Marital status				
Married	1798 (70.3)	959 (72.9)	839 (67.6)	.01
Single	266 (10.4)	124 (9.4)	142 (11.4)	
Other	492 (19.3)	232 (17.6)	260 (21.0)	
Comorbidities in prior year				
Hypertension	2114 (82.7)	1133 (86.2)	981 (79.0)	<.001
Hyperlipidemia	2336 (91.4)	1214 (92.3)	1122 (90.4)	.09
Diabetes	1173 (45.9)	614 (46.7)	559 (45.0)	.40
HF	1356 (53.1)	725 (55.1)	631 (50.8)	.03
MI	1364 (53.4)	686 (52.2)	678 (54.6)	.21
Stroke	415 (16.2)	243 (18.5)	172 (13.9)	.002
Obesity	1091 (42.7)	528 (40.2)	563 (45.4)	.008
Chronic kidney disease	743 (29.1)	413 (31.4)	330 (26.6)	.01
Depression	45 (1.8)	31 (2.4)	14 (1.1)	.02
Charlson Comorbidity Index				
0	99 (3.9)	46 (3.5)	53 (4.3)	.002
1	366 (14.3)	157 (11.9)	209 (16.8)	
2	482 (18.9)	239 (18.2)	243 (19.6)	
3	413 (16.2)	224 (17.0)	189 (15.2)	
≥4	1196 (46.8)	649 (49.4)	547 (44.1)	
Smoking status				
Ever	1113 (43.5)	559 (42.5)	554 (44.6)	.28
Never	1443 (54.5)	756 (57.5)	687 (55.4)	
Healthcare utilization in prior year				
Hospitalization	2069 (80.9)	1128 (85.8)	941 (75.8)	<.001
Emergency department visit	1708 (66.8)	878 (66.8)	830 (66.9)	.95
Urgent care visit	989 (38.7)	504 (38.3)	485 (39.1)	.70
Cardiovascular risk factor control in prior year				
Blood pressure, mm Hg				
Systolic <140	2072 (81.1)	1083 (82.4)	989 (79.7)	.09
Diastolic <90	2474 (96.8)	1281 (97.4)	1193 (96.1)	.07
Laboratory values in prior year				
LDL cholesterol <100 mg/dL	1734 (67.8)	908 (69)	826 (66.6)	.18
HbA_1C_ <7%	2038 (79.7)	1062 (80.8)	976 (78.6)	.18
BMI <25	543 (21.2)	343 (26.1)	200 (16.1)	<.001
Reason for referral to CR				
Nonsurgical				
MI	574 (22.5)	252 (19.2)	322 (25.9)	<.001
PCI	331 (12.9)	154 (11.7)	177 (14.3)	
Angina	87 (3.4)	38 (2.9)	49 (3.9)	
HF	390 (15.3)	159 (12.1)	231 (18.6)	
Surgical				
Heart transplant	14 (0.5)	14 (1.1)	0 (0)	
CABG	647 (25.3)	385 (29.3)	262 (21.1)	
Valve repair or replacement	513 (20.1)	313 (23.8)	200 (16.1)	
Driving distance from home to medical center, mean (SD), miles	11.9 (11.7)	10.8 (10.1)	13.0 (13.2)	.007
Total exercise sessions during CR				
1-12	688 (26.9)	454 (34.5)	234 (18.8)	<.001
12-24	591 (23.1)	276 (21.0)	315 (25.4)	
24-36	646 (25.3)	296 (22.5)	350 (28.2)	
≥36	631 (24.7)	289 (22.0)	342 (27.6)	

Abbreviations: BMI, body mass index (calculated as weight in kilograms divided by height in meters squared); CABG, coronary artery bypass graft surgery; CBCR, center-based cardiac rehabilitation; CR, cardiac rehabilitation; HbA_1c_, hemoglobin A_1c_; HBCR, home-based cardiac rehabilitation; HF, heart failure; LDL, low-density lipoprotein; MI, myocardial infarction; PCI, percutaneous coronary intervention.

SI conversion factors: To convert HbA_1c_ to proportion of total hemoglobin, multiply by 0.01; LDL cholesterol to millimoles per liter, multiply by 0.0259.

^a^ Includes individuals listed as American Indian or Alaska Native, more than 1 race, other race, or unknown race.

Compared with patients who participated in CBCR, patients who participated in HBCR were younger, more likely to Black or Hispanic, and less likely to be White (Table 1). They had lower median household income and were less likely to be married (Table 1). They had fewer comorbidities; for example, patients who participated in HBCR were less likely than patients who participated in CBCR to have hypertension, CHF, history of stroke, CKD, or major depression, but they were more likely to have obesity (Table 1). Patients who received HBCR were less likely to have been hospitalized in the year prior to their CR qualifying event. They were also less likely to have experienced cardiac surgical treatment as their CR-eligible qualifying event (Table 1). Additionally, patients who participated in HBCR lived farther from their home medical center but were more likely to adhere to their CR program, defined as completing at least 36 sessions. Through IPTW, the baseline patient characteristics were balanced between groups (eTable 2 in the Supplement).

### Primary and Secondary Outcomes

During the 12-month follow-up, there were fewer all-cause hospitalizations among patients who participated in HBCR compared with those who participated in CBCR (184 participants [14.8%] vs 238 participants [18.1%]; OR, 0.79; 95% CI, 0.64-0.97; *P* = .03) (Table 2). There was no statistically significant difference between participants in HBCR vs CBCR in 30- or 90-day all-cause or CVD hospitalization (Table 2). Additionally, there was no statistically significant difference between the 2 groups in adherence to β-blockers (OR, 1.18; 95% CI, 0.98-1.42) or statins (OR, 1.02; 95% CI, 0.84-1.25) or control of blood pressure (OR, 0.98; 95% CI, 0.81-1.17), LDL-cholesterol (OR, 0.98; 95% CI, 0.81-1.20), or HbA_1c_ (OR, 0.98; 95% CI, 0.82-1.18) at 12 months (Table 2 and Figure; eTable 3 in the Supplement). In interaction analyses by sex, race and ethnicity, and Charlson Comorbidity Index, no statistically significant interactions were present (eTable 4 in the Supplement).

**Table 2.** Hospitalization Events, Adherence to Medication, and Cardiovascular Risk Factor Control for CBCR and HBCR Before and After Inverse Probability of Treatment Weighting

Outcomes	No. (%)			*P* value
	Total (N = 2556)	CBCR (n = 1315)	HBCR (n = 1241)	
**Unweighted**				
All-cause hospitalization				
30-d	49 (1.9)	31 (2.4)	18 (1.5)	.09
90-d	132 (5.2)	79 (6)	53 (4.3)	.05
12-mo	425 (16.6)	244 (18.6)	181 (14.6)	.01
Cardiovascular-related hospitalization				
30-d	24 (0.9)	15 (1.1)	9 (0.7)	.28
90-d	67 (2.6)	42 (3.2)	25 (2.0)	.06
12-mo	217 (8.5)	121 (9.2)	96 (7.7)	.18
Statin adherence, PDC >80	1843 (79)	947 (79)	896 (78.9)	.98
β-blocker adherence, PDC >80	1752 (74.8)	870 (73.3)	882 (76.4)	.08
Cardiovascular risk factor control				
Blood pressure, mm Hg				
Systolic <140	1962 (76.8)	1002 (76.2)	960 (77.4)	.49
Diastolic <90	2427 (95.0)	1248 (94.9)	1179 (95.0)	.91
LDL cholesterol <100 mg/dL	2091 (81.8)	1077 (81.9)	1014 (81.7)	.90
HbA_1c_ <7%	1932 (75.6)	1001 (76.1)	931 (75)	.52
BMI <25	519 (20.3)	319 (24.3)	200 (16.1)	<.001
**Weighted**				
All-cause hospitalization				
30-d	51 (2.0)	31(2.4)	20 (1.6)	.16
90-d	132 (5.2)	79 (6.0)	53 (4.3)	.06
12-mo	422 (16.5)	238 (18.1)	184 (14.8)	.03
Cardiovascular-related hospitalization				
30-d	24 (0.9)	15 (1.2)	9 (0.7)	.22
90-d	65 (2.5)	41(3.1)	23 (1.9)	.04
12-mo	214 (8.4)	121 (9.2)	94 (7.6)	.41
Statin adherence, PDC >80	1844 (78.8)	941 (78.6)	903 (79.0)	.81
β-blocker adherence, PDC >80	1748 (74.9)	866 (73.4)	882 (76.4)	.09
Cardiovascular risk factor control				
Blood pressure, mm Hg				
Systolic <140	1957 (76.6)	1010 (76.8)	948 (76.4)	.79
Diastolic <90	2430 (95.1)	1248 (94.9)	1182 (95.3)	.65
LDL cholesterol <100 mg/dL	2089 (81.7)	1075 (81.8)	1012 (81.5)	.87
HbA_1c_ <7%	1922 (75.2)	990 (75.3)	930 (75.0)	.84
BMI <25	519 (20.3)	273 (20.7)	244 (19.6)	.49

Abbreviations: BMI, body mass index (calculated as weight in kilograms divided by height in meters squared); CBCR, center-based cardiac rehabilitation; HbA_1c_, hemoglobin A_1c_; HBCR, home-based cardiac rehabilitation; LDL, low-density lipoprotein; PDC, proportion of days covered.

SI conversion factors: To convert HbA_1c_ to proportion of total hemoglobin, multiply by 0.01; LDL cholesterol to millimoles per liter, multiply by 0.0259.

**Figure.**
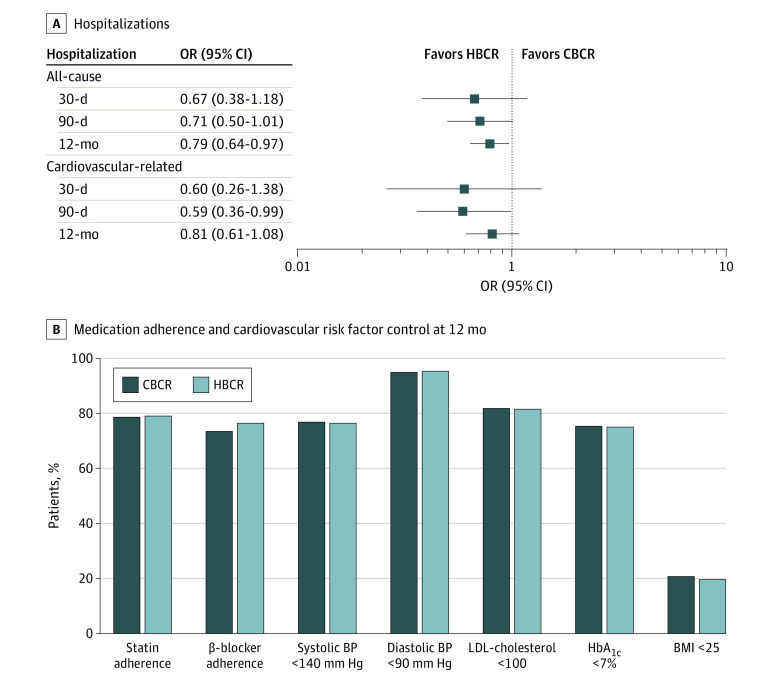
Home-Based Cardiac Rehabilitation (HBCR) vs Center-Based Cardiac Rehabilitation (CBCR) BMI indicates body mass index (calculated as weight in kilograms divided by height in meters squared); BP, blood pressure; HbA_1c_, hemoglobin A_1c_; LDL, low-density lipoprotein; and OR, odds ratio. To convert HbA_1c_ to proportion of total hemoglobin, multiply by 0.01; LDL-cholesterol to millimoles per liter, multiply by 0.0259.

## Discussion

This cohort study found that compared with CBCR, participation in HBCR was associated with fewer all-cause hospitalization at 12 months. Our study size of 2556 KPSC patients who participated in CR (1241 participated in HBCR and 1315 participated in CBCR) is the largest demographically diverse and medically complex cohort study of CR in the literature, to our knowledge. Additionally, to our knowledge, we are the first study to report superior clinical outcomes in HBCR compared with CBCR and the first to examine hospitalizations as the primary outcome. In general, most prior HBCR vs CBCR comparison studies have focused on patient-centered outcomes, such as cardiorespiratory fitness,^38^ quality of life,^39^ or patient satisfaction,^39^ as the primary outcome measure. For instance, in the most recent Cochrane meta-analysis^1^ of 23 HBCR vs CBCR RCTs, all trials reported on cardiorespiratory fitness, 11 trials reported on mortality (and the strength of this evidence was considered poor), and only 5 trials reported on hospitalizations, all exclusively as a secondary outcome. This study expands the evidence base regarding the association of HBCR participation with subsequent hospitalization.

Additional novel aspects of our study include enrollment of a well-characterized, demographically diverse population of patients, with comprehensive longitudinal data and including previously understudied populations, namely women and patients who were Asian or Pacific Islander, Black, or Hispanic. In contrast, the most recent HBCR Cochrane meta-analysis^1^ enrolled less than 20% women overall (23 total trials and 4 trials excluding women altogether). Additionally, with regards to race and ethnicity, most trials within the 2017 meta-analysis^1^ (19 of 23 trials) did not report race and ethnicity, and among the 4 remaining trials that did report race and ethnicity, the study populations were all predominantly White.

Second, our analysis includes a large population of patients who are medically complex. We used the Charlson Comorbidity Index to evaluate medical complexity. The index is widely used as a 12-month mortality risk indicator^40^ and has been previously validated among patients with CVD.^41,42^ Nearly half of patients in this study had a Charlson Comorbidity Index score of 4 or higher, which is suggestive of moderate to high risk of 12-month mortality. Although prior HBCR vs CBCR studies have reported outcomes among patients with higher risk, there has been considerable heterogeneity regarding the risk measurement use and small sample sizes of patients with the highest risk. This has led to the viewpoint that HBCR studies often are restricted to a minority of potential patients—those at lower risk and with fewer comorbidities.^43^ For instance, a study by Bravo-Escobar et al^38^ sought to examine effectiveness and safety of HBCR in patients with coronary artery disease in Malaga, Spain. They conducted an RCT of 28 patients with high risk (14 CBCR patients and 14 HBCR patients) who had undergone CABG or revascularization with PCI. They defined risk according to Spanish Cardiology Society guidelines and found no statistically significant differences in study outcomes (ie, exercise time defined as metabolic equivalents achieved during the exertion test and the recovery rate in the first minute).^38^ In contrast, our study presents findings from a large population of patients who were medically complex and higher risk using a well-established risk measurement instrument that has been validated in patients with CVD. Taken together, our study adds to the HBCR outcomes literature by reporting much needed data on previously underrepresented populations, including women, racial and ethnic minority groups, and patients with multiple comorbidities.

Finally, our study found that there was a significant difference in driving distance or location of patient relative to their home medical center. Specifically, HBCR patients lived farther from their home medical center but demonstrated better program adherence compared with CBCR patients. This finding is not surprising, given that HBCR allows for the enrollment of patients who are unable to travel, a common reason given for lack of participation in CBCR.^44,45,46^ However, this finding is important, given the recent nationwide increase in referrals to HBCR that occurred after the unexpected widespread closure of CBCR programs following the onset of the COVID-19 pandemic and the subsequent call from medical societies to continue to increase participation in HBCR in all eligible patients in the current pandemic era.^47,48^

### Limitations

Our study has some limitations. First, given the retrospective observational study design, we applied statistical techniques to balance known confounders; however, we cannot account for unknown confounders. Second, the decision to refer patients to HBCR vs CBCR was at the sole discretion of the treating cardiologist. There may have been physician referral bias, which may have impacted outcomes.^49^ Future research is needed with a randomized design to address these limitations. Third, although we reported the number of sessions completed by study participants, our study analyzed patient outcomes using the standard definition for CR participation that has been used in prior observational studies (specifically, participation in ≥1 CR session). As such, we are unable to determine if the “dose” or amount of CR is independently associated with outcomes. Although data from CBCR literature has demonstrated a reproducible dose-response association between CR session attendance and reduced risk of major adverse cardiovascular events,^50,51,52,53^ to our knowledge, no research has established this association in the HBCR setting. Fourth, our study reports outcomes in the pre–COVID-19 pandemic period, thus allowing for comparison of outcomes without confounding of health system changes that occurred following the onset of the pandemic. It is unknown whether our findings can be applied to a postpandemic era. Fifth, although the KPSC HBCR used a multidisciplinary, comprehensive format, similar in structure to most HBCR programs included in the Cochrane Collaborative metanalyses, our HBCR program also included the use of a wearable device. A 2019 meta-analysis^54^ of 9 HBCR studies involving 1352 participants found that use of a wearable device was associated with improved outcomes compared with HBCR without a wearable device. This may limit generalizability of our findings Additionally, our study analyzes patients within an integrated health system, where the insurance plan, hospitals, pharmacy, and medical practitioners are integrated to create a system for coordinated and comprehensive health care. Therefore, these results may be less generalizable to individuals in less-integrated settings or among uninsured individuals. Despite these limitations, strengths of the current study include the use of a large, diverse, and well-characterized population of KPSC patients with comprehensive EMR and pharmacy records to examine baseline comorbidities and 12-month outcomes.

## Conclusions

In this demographically diverse cohort study that included patients who were medically complex with high risk, we found that participation in HBCR was associated with fewer hospitalizations at 12 months compared with participation in CBCR. Our findings strengthen the evidence base for the use of HBCR as a viable alternative to CBCR in all patients, including previously understudied key subgroups.

## Notes

Supplement. **eTable 1.** Description of Kaiser Permanente Southern California (KPSC) Cardiac Rehabilitation Programs **eTable 2.** Demographic and Clinical Characteristics of Patients Participating in Center-Based and Home-Based Cardiac Rehabilitation Before and After IPTW **eTable 3.** Adjusted Odds Ratios for Hospitalization Events, Adherence to Medication, and Cardiovascular Risk Factor Control Comparing Home-Based and Center-Based Cardiac Rehabilitation Before and After IPTW **eTable 4.** Tests of Interaction for Subgroup Analyses Comparing 12-Month All-Cause Hospitalization in Home-Based and Center-Based Cardiac Rehabilitation Groups **eFigure.** Derivation of the Study Cohort
