# Supplementary material for: Comparison of Home-Based vs Center-Based Cardiac Rehabilitation in Hospitalization, Medication Adherence, and Risk Factor Control Among Patients With Cardiovascular Disease
Source: JAMA Netw Open. 2022 Aug 25;5(8):e2228720. doi: 10.1001/jamanetworkopen.2022.28720 (PMC9412228; doi:10.1001/jamanetworkopen.2022.28720)
Supplement: Supplement. — eTable 1. Description of Kaiser Permanente Southern California (KPSC) Cardiac Rehabilitation Programs eTable 2. Demographic and Clinical Characteristics of Patients Participating in Center-Based and Home-Based Cardiac Rehabilitation Before and After IPTW eTable 3. Adjusted Odds Ratios for Hospitalization Events, Adherence to Medication, and Cardiovascular Risk Factor Control Comparing Home-Based and Center-Based Cardiac Rehabilitation Before and After IPTW eTable 4. Tests of Interaction for Subgroup Analyses Comparing 12-Month All-Cause Hospitalization in Home-Based and Center-Based Cardiac Rehabilitation Groups eFigure. Derivation of the Study Cohort [file jamanetwopen-e2228720-s001.pdf]

Supplemental Online Content

Nkonde-Price C, Reynolds K, Najem M, et al. Comparison of home-based vs center-based cardiac rehabilitation in hospitalization, medication adherence, and risk factor control among patients with cardiovascular disease. *JAMA Netw Open*. 2022;5(8):e2228720. doi:10.1001/jamanetworkopen.2022.28720

eTable 1. Description of Kaiser Permanente Southern California (KPSC) Cardiac Rehabilitation Programs
eTable 2. Demographic and Clinical Characteristics of Patients Participating in Center-Based and Home-Based Cardiac Rehabilitation Before and After IPTW
eTable 3. Adjusted Odds Ratios for Hospitalization Events, Adherence to Medication, and Cardiovascular Risk Factor Control Comparing Home-Based and Center-Based Cardiac Rehabilitation Before and After IPTW
eTable 4. Tests of Interaction for Subgroup Analyses Comparing 12-Month All-Cause Hospitalization in Home-Based and Center-Based Cardiac Rehabilitation Groups
eFigure. Derivation of the Study Cohort

This supplemental material has been provided by the authors to give readers additional information about their work.

© 2022 Nkonde-Price C et al. *JAMA Network Open.*

eTable 1. Description of Kaiser Permanente Southern California (KPSC) Cardiac Rehabilitation Programs

CR Program Description	Exercise Prescription	Exercise Documentation
**Description of HBCR:**<br>Patients are referred to an 8-week technology enabled home-based CR program. At the beginning of the program, patients attend an initial in-person visit at a KPSC outpatient Cardiology Department where eligibility criteria are verified and the patient is supplied with a modified Samsung smartwatch and HeartWise mobile application that tracks exercise, medication use, and symptoms. During the initial in-person visit, the case manager and patient discuss overall program goals based on the patient's current situation and results of a 6-minute walking test. The exercise regimen consists of 36 sessions (30-minute sessions, 5 days a week). The smartwatch collects the following data: pulse, number of steps, exercise activity, and inactivity and transmits this to the EMR<br><br>**Components:** Exercise(unsupervised), education (printed, online videos and in person classes) psychosocial<br><br>**Program Type**: Comprehensive<br><br>**Length of exercise session:** 30 minute/session<br><br>**Frequency/ no sessions:** 5 sessions weekly<br><br>**Total duration:** 8 weeks<br><br>**Intermittent nurse or exercise specialist telephone support:**<br>HBCR patients were telephoned every week by CR nurse to monitor progress, revise the exercise prescription if necessary and provide behavioral coaching and support, depression screening and referral for psychological support and health education. Exercise logs reviewed weekly.<br><br>**Co-interventions:** Referral to additional health education classes at local KPSC Medical Center for Health Living (Heart Healthy Dietary Advice, Stress Management, Smoking Cessation)<br><br>**Technology Enabled:** Mobile phone application (Heartwise) linked to a wearable smartwatch (modified Samsung smartwatch)	**HBCR:**<br>Exercise prescription was determined by the referring Cardiologist.	**HBCR**: Every exercise session completed by the patient was recorded by the Smartwatch and electronically transmitted via secure cloud and recorded in EMR.

© 2022 Nkonde-Price C et al. *JAMA Network Open.*

CR Program Description	Exercise Prescription	Exercise Documentation
**Description of CBCR:**<br><br>Patients are referred to one of 13 outpatient cardiac rehabilitation centers that are associated with their home medical center (defined as the Kaiser Permanente Medical Center closet to their zip code). They attend 3 x 60min supervised exercise sessions per week for a total of 36 sessions. There is also a multidisciplinary educational program available to patient (to be completed either before or after each exercise session that consists of nutrition counseling and sessions to improve a patient's overall cardiovascular health and lifestyle. All CBCR programs accredited by the American Association of Cardiovascular and Pulmonary Rehabilitation (AACVPR).<br><br>**Components:** Exercise (supervised) and education<br><br>**Program Type:** Comprehensive<br><br>**Length of exercise session:** 30 minute/session<br><br>**Frequency/ no sessions:** 3 sessions weekly<br><br>**Total duration:** 12 weeks<br><br>**Co-interventions:** Not Reported	**CBCR:**<br>Exercise prescription was determined by the referring Cardiologist	**CBCR:**<br>Exercise sessions were recorded and documented in either a paper medical record or electronic medical record depending on the local Center policy

© 2022 Nkonde-Price C et al. *JAMA Network Open.*

eTable 2. Demographic and Clinical Characteristics of Patients Participating in Center-Based and Home-Based Cardiac Rehabilitation Before and After IPTW

	Unweighted				Weighted			
	**CBCR N=1315**	**HBCR N=1241**	**p-value**	**Standardized difference**	**CBCR N=1315**	**HBCR N=1241**	**p-value**	**Standardized difference**
**Age group, years**			<0.0001	0.24			1.00	0.003
< 45	33 (2.5%)	66 (5.3%)			51 (3.9%)	49 (3.9%)		
45 - 65	423 (32.2%)	495 (39.9%)			472 (35.9%)	446 (35.9%)		
≥ 65	859 (65.3%)	680 (54.8%)			792 (60.2%)	746 (60.1%)		
**Sex**			0.17	0.05			0.99	0.0004
Women	372 (28.3%)	382 (30.8%)			386 (29.4%)	364 (29.3%)		
Men	943 (71.7%)	859 (69.2%)			929 (70.6%)	877 (70.7%)		
**Race/Ethnicity**			0.13	0.09			1.00	0.006
Asian/ Pacific Islander	158 (12%)	131 (10.6%)			147 (11.2%)	138 (11.1%)		
Black	90 (6.8%)	103 (8.3%)			101 (7.7%)	97 (7.8%)		
Hispanic	288 (21.9%)	323 (26%)			321 (24.4%)	302 (24.3%)		
White	760 (57.8%)	659 (53.1%)			722 (54.9%)	682 (55.0%)		
Other	19 (1.4%)	25 (2%)			24 (1.8%)	22 (1.8%)		
**Language, Interpreter Needed**	82 (6.2%)	60 (4.8%)	0.12	0.06	75 (5.7%)	74 (6.0%)	0.79	0.011
**Neighborhood median household income**			0.048	0.10			0.99	0.005
<= $45,000	135 (10.3%)	133 (10.7%)			132 (10.1%)	126 (10.2%)		
$45,001 - $80,000	514 (39.1%)	539 (43.4%)			547 (41.6%)	518 (41.7%)		
**> $**80,000	666 (50.6%)	569 (45.9%)			636 (48.4%)	597 (48.1%)		
**Marital status**			0.01	0.12			0.99	0.005
Married	959 (72.9%)	839 (67.6%)			923 (70.2%)	869 (70.1%)		
	**CBCR N=1315**	**HBCR N=1241**			**CBCR N=1315**	**HBCR N=1241**		

© 2022 Nkonde-Price C et al. *JAMA Network Open.*

Single	124 (9.4%)	142 (11.4%)			142 (10.8%)	133 (10.7%)		
Others	232 (17.6%)	260 (21%)			250 (19%)	239 (19.2%)		
**Comorbidities in prior year**	**CBCR N=1315**	**HBCR N=1241**	**p-value**	**Standardized difference**	**CBCR N=1315**	**HBCR N=1241**	**p-value**	**Standardized difference**
Hypertension	1133 (86.2%)	981 (79%)	<0.0001	0.19	1090 (82.9%)	1029 (82.9%)	0.98	0.001
Hyperlipidemia	1214 (92.3%)	1122 (90.4%)	0.09	0.07	1203 (91.5%)	1135 (91.5%)	0.96	0.002
Diabetes	614 (46.7%)	559 (45.0%)	0.40	0.03	613 (46.6%)	580 (46.8%)	0.93	0.003
Heart failure	725 (55.1%)	631 (50.8%)	0.03	0.09	702 (53.4%)	662 (53.4%)	0.99	0.001
Myocardial Infarction	686 (52.2%)	678 (54.6%)	0.21	0.05	703 (53.4%)	663 (53.5%)	0.99	0.001
Stroke	243 (18.5%)	172 (13.9%)	0.002	0.13	217 (16.5%)	204 (16.5%)	0.96	0.002
Chronic kidney disease	413 (31.4%)	330 (26.6%)	0.01	0.11	383 (29.1%)	363 (29.3%)	0.94	0.003
Depression	31 (2.4%)	14 (1.1%)	0.02	0.09	24 (1.8%)	20 (1.6%)	0.76	0.001
**Charlson comorbidity index**			0.0007	0.16			1.00	0.006
≤ 1	203 (15.4%)	262 (21.1%)			233 (17.7%)	222 (17.9%)		
2	239 (18.2%)	243 (19.6%)			244 (18.6%)	229 (18.4%)		
3	224 (17%)	189 (15.2%)			215 (16.4%)	203 (16.3%)		
≥ 4	649 (49.4%)	547 (44.1%)			623 (47.4%)	588 (47.4%)		
**Ever smoker**	559 (42.5%)	554 (44.6%)	0.28	0.04	571 (43.4%)	543 (43.7%)	0.88	0.006
**Hospitalization in prior year**	1128 (85.8%)	941 (75.8%)	<0.0001	0.25	1069 (81.3%)	1002 (80.7%)	0.69	0.016
**Cardiovascular risk factor control in prior year**								
Systolic blood pressure, <140 mm Hg	1083 (82.4%)	989 (79.7%)	0.09	0.07	1061 (80.7%)	1005 (81.0%)	0.85	0.008
	**CBCR N=1315**	**HBCR N=1241**	**p-value**	**Standardized difference**	**CBCR N=1315**	**HBCR N=1241**	**p-value**	**Standardized difference**
Diastolic blood pressure, <90 mm Hg	1281 (97.4%)	1193 (96.1%)	0.07	0.07	1274 (96.9%)	1202 (96.9%)	0.99	0.001

© 2022 Nkonde-Price C et al. *JAMA Network Open.*

LDL-cholesterol, <100 mg/dL	908 (69%)	826 (66.6%)	0.18	0.05	886 (67.4%)	838 (67.5%)	0.94	0.003
HbA1c, <7%	1062 (80.8%)	976 (78.6%)	0.18	0.05	1039 (79.0%)	981 (79.1%)	0.98	0.001
Body mass index, <25 kg/m^2	343 (26.1%)	200 (16.1%)	<0.0001	0.25	277 (21.1%)	256 (20.6%)	0.78	0.011
**Referral to CR due to Cardiothoracic Surgery**	712 (54.1%)	462 (37.2%)	<0.0001	0.34	601 (45.7%)	562 (45.3%)	0.82	0.009
**Total exercise sessions during CR**			0.001	0.130			0.95	0.002
< 36	1026 (78%)	899 (72.4%)			987 (75.1%)	933 (75.2%)		
≥ 36	289 (22%)	342 (27.6%)			328 (24.9%)	308 (24.8%)		

IPTW = inverse probability of treatment weighting; CBCR = center based cardiac rehabilitation; HBCR = home based cardiac rehabilitation; AMI = acute myocardial infarction. CR = cardiac rehabilitation; Other race = American Indian and Alaska Native, More than one Race or Unknown Race

© 2022 Nkonde-Price C et al. *JAMA Network Open.*

eTable 3. Adjusted Odds Ratios for Hospitalization Events, Adherence to Medication, and Cardiovascular Risk Factor Control Comparing Home-Based and Center-Based Cardiac Rehabilitation Before and After IPTW

	HBCR vs CBCR			
	**Unweighted OR (95% CI)**	**p-value**	**Weighted OR (95% CI)**	**p-value**
**All-cause hospitalization**				
30-day	0.61 (0.34-1.10)	0.10	0.67 (0.38-1.18)	0.16
90-day	0.70 (0.49-1.00)	0.05	0.70 (0.50-1.01)	0.06
12 Month	0.75 (0.61-0.93)	0.01	0.79 (0.64-0.97)	0.03
**Cardiovascular-related hospitalization**				
30-day	0.63 (0.28-1.45)	0.28	0.60 (0.26-1.38)	0.22
90-day	0.62 (0.38-1.03)	0.06	0.59 (0.36-0.99)	0.04
12 Month	0.83 (0.63-1.10)	0.18	0.81 (0.61-1.08)	0.14
**Statin adherence**	0.99 (0.82-1.21)	0.98	1.02 (0.84-1.25)	0.81
**Beta blocker adherence**	1.18 (0.98-1.43)	0.08	1.18 (0.98-1.42)	0.09
**Cardiovascular risk factor control**				
Systolic blood pressure, <140 mm Hg	1.07 (0.89-1.28)	0.49	0.98 (0.81-1.17)	0.79
Diastolic blood pressure <90 mm Hg	1.02 (0.72-1.46)	0.91	1.09 (0.76-1.56)	0.65
LDL-cholesterol <100 mg/dL	0.99 (0.81-1.21)	0.90	0.98 (0.81-1.20)	0.87
HbA1c <7 %	0.94 (0.79-1.13)	0.52	0.98 (0.82-1.18)	0.84
Body mass index, <25 kg/m^2	0.06 (0.49-0.73)	<0.0001	0.93 (0.77-1.13)	0.49

IPTW = inverse probability of treatment weighting; CBCR = center based cardiac rehabilitation; HBCR = home based cardiac rehabilitation

© 2022 Nkonde-Price C et al. *JAMA Network Open.*

eTable 4. Tests of Interaction for Subgroup Analyses Comparing 12-Month All-Cause Hospitalization in Home-Based and Center-Based Cardiac Rehabilitation Groups

Dependent Variable (Outcome)	Independent Variable	Unweighted (Univariate)		Weighted		Multivariate[a]	
		Beta Coefficient	p-value	Beta Coefficient	p-value	Beta Coefficient	p-value
12-month All-cause Hospitalization	Model 1:						
	CR Program	-0.01	0.95	-0.01	0.96	0.00	0.99
	Sex	0.13	0.43	0.10	0.53	0.21	0.23
	CR Program*Sex	-0.40	0.09	-0.33	0.16	-0.38	0.11
	Model 2:						
	CR Program	-0.49	0.02	-0.53	0.01	-0.47	0.03
	Race	-0.14	0.04	-0.18	0.01	-0.11	0.12
	CR Program*Race	0.11	0.26	0.15	0.13	0.10	0.31
	Model 3:						
	CR Program	-0.30	0.08	-0.20	0.25	-0.30	0.09
	CCI	0.80	<.0001	0.91	<.0001	0.46	0.02
	CR Program*CCI	0.08	0.73	-0.07	0.75	0.07	0.75

CR= cardiac rehabilitation CCI= Charlson Co-morbidity Index

Logistic Regression
a Model 1: adjusted for age, median household income, race, marital status, need interpreter, comorbidities (CKD, DM, HLD, HTN, congestive heart failure, MI, Stroke, depression, overweight, Charlson co-morbidity index), smoking status, hospital admission prior to participating rehab program, reason of referral to rehab program, number of rehab session completed.

Model 2: adjusted for age, median household income, gender, marital status, need interpreter, comorbidities (CKD, DM, HLD, HTN, congestive heart failure, MI, Stroke, depression, overweight, Charlson co-morbidity index), smoking status, hospital admission prior to participating rehab program, reason of referral to rehab program, number of rehab session completed.

Model 3: adjusted for age, median household income, race, gender, marital status, need interpreter, comorbidities (CKD, DM, HLD, HTN, congestive heart failure, MI, Stroke, depression, overweight), smoking status, hospital admission prior to participating rehab program, reason of referral to rehab program, number of rehab session completed.

© 2022 Nkonde-Price C et al. *JAMA Network Open.*

eFigure 1. Derivation of the Study Cohort

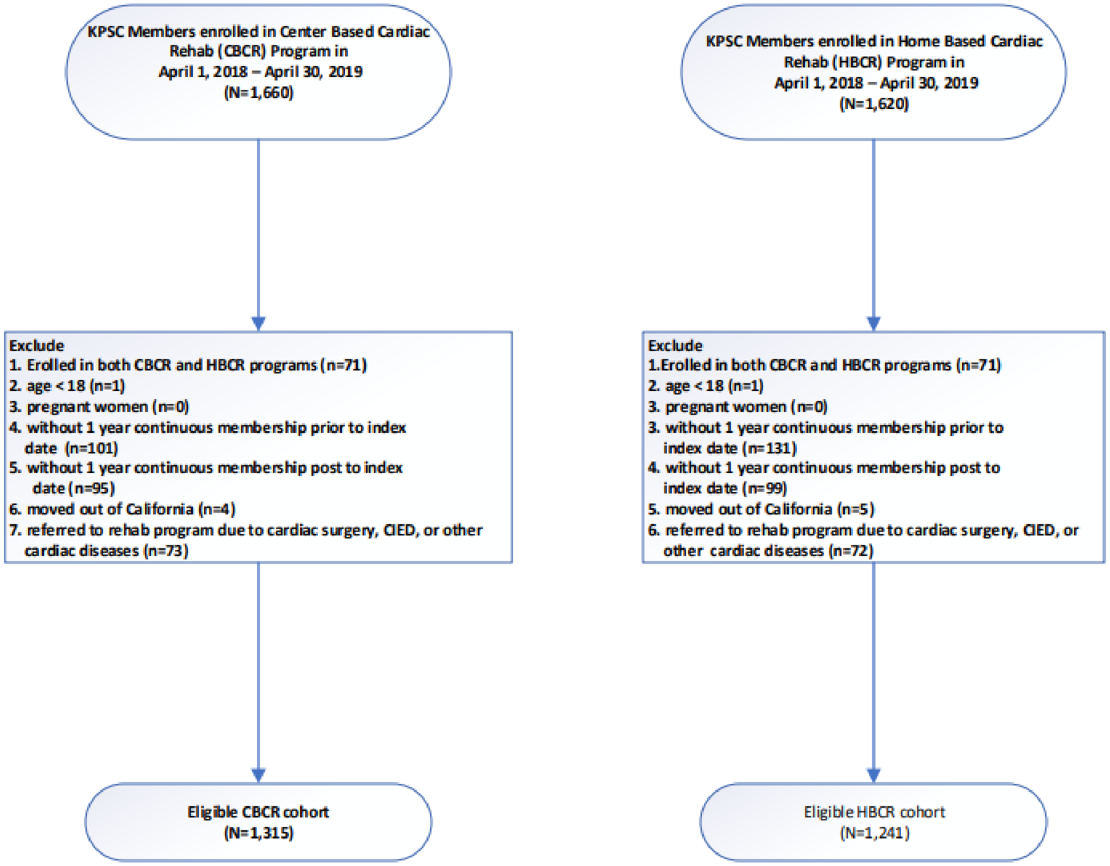

© 2022 Nkonde-Price C et al. *JAMA Network Open.*
